# Workplace Bullying Among Junior Doctors in Malaysia: A Multicentre Cross-Sectional Study

**DOI:** 10.21315/mjms2021.28.2.13

**Published:** 2021-04-21

**Authors:** Ely Zarina Samsudin, Marzuki Isahak, Sanjay Rampal, Ismail Rosnah, Mohd Idzwan Zakaria

**Affiliations:** 1 Department of Public Health Medicine, Faculty of Medicine, Universiti Teknologi MARA, Selangor, Malaysia; 2 Department of Social and Preventive Medicine, Faculty of Medicine, University of Malaya, Kuala Lumpur, Malaysia; 3 Department of Community Health, Faculty of Medicine, Universiti Kebangsaan Malaysia, Bangi, Selangor, Malaysia; 4 Dean’s Office, Faculty of Medicine, University of Malaya, Kuala Lumpur, Malaysia

**Keywords:** workplace bullying, junior doctors, prevalence, associated factors, Occupational Safety and Health, psychosocial hazard

## Abstract

**Background:**

Research suggests that junior doctors often experience workplace bullying, which may have adverse impacts on medical training and delivery of quality healthcare. However, evidence among local population has not been established. The present study aims to examine the prevalence of workplace bullying among Malaysian junior doctors and explore its associated sociodemographic and employment factors.

**Methods:**

A multicentre cross-sectional study was conducted in 12 government hospitals accredited for housemanship training within the central zone of Malaysia. The study included a total of 1,074 house officers who had been working for at least 6 months in various housemanship rotations. The Negative Acts Questionnaire-Revised (NAQ-R*)* was used to examine workplace bullying.

**Results:**

The 6-month prevalence of workplace bullying among study participants was 13%. Work-related bullying such as ‘being ordered to do work below your level of competence’, person-related bullying such as ‘being humiliated or ridiculed in connection with your work’, and physically intimidating bullying such as ‘being shouted at or being the target of spontaneous anger’ were commonly reported by study participants. Medical officers were reported to be the commonest perpetrators of negative actions at the workplace. Study participants who graduated from Eastern European medical schools (adjusted odds ratio [AOR] 2.27; 95% confidence interval [CI]: 1.27, 4.07) and worked in surgical-based rotation (AOR 1.83; 95% CI: 1.13, 2.97) had higher odds of bullying compared to those who graduated from local medical schools and worked in medical-based rotation, whereas study participants with good English proficiency (AOR 0.14; 95% CI: 0.02, 0.94) had lower odds of bullying compared to those with poor English proficiency.

**Conclusion:**

The present study shows that workplace bullying is prevalent among Malaysian junior doctors. Considering the gravity of its consequences, impactful strategies should be developed and implemented promptly in order to tackle this serious occupational hazard.

## Introduction

Workplace bullying, a phenomenon that is also labelled as workplace mobbing, harassment, aggression, emotional abuse and victimisation (1), has emerged as a significant workplace health and safety problem. Defined as ‘situations where an employee is persistently exposed to negative and aggressive behaviours at work primarily of a psychological nature with the effect of humiliating, intimidating, frightening or punishing the target’ (2), it is a problem that has been shown to exist worldwide (3–4). Within the healthcare industry, many cases of bullying experienced by doctors are reported to be perpetrated by others in a pecking order of seniority (5). Indeed, studies have demonstrated that 30% to 95% of junior doctors across the globe report being bullied at work (6). This has been suggested to be due to the traditional hierarchical structures of hospitals and gruelling medical training, which produces a culture in which bullying is not only unchallenged, but perceived as a ‘functional educational tool’ (7–8). Further compounding the problem is the culture of silence in which only 12% of junior doctors report experiences of abuse to a supervisor, for fear of repercussions in reporting mistreatment (9). Thus, such incidences are allowed to persist and perpetuate, causing ‘bullying’ to become a learned behaviour that gives rise to a legacy of abuse in medicine (8–11).

In Malaysia, numerous newspaper articles have highlighted cases of workplace bullying among junior doctors over the past few years (12–15). These incidences were shown to lead to detrimental repercussions for the targets of bullying, with some developing depression, quitting housemanship and even considering suicide (12–15). Indeed, a considerable body of literature has established that junior doctors’ exposure to workplace bullying have resulted in undesirable health and work outcomes, such as mental strain, job dissatisfaction and burnout (16–20). Disconcertingly, bullied junior doctors have also been reported to be more likely to make serious or potentially serious medical errors and had higher frequency of accidents compared to non-bullied peers (17, 21). These effects may not only negatively impact their training but may also hinder the delivery of safe and high-quality patient care. Despite the above concerns, to date, no studies have been published to examine the occurrence of workplace bullying among local junior doctor population. Correspondingly, the present study attempts to determine the prevalence of workplace bullying among Malaysian junior doctors and explore the sociodemographic and employment factors associated with it.

## Methods

### Sample

The terminology for junior doctors varies across the globe, including terms such as ‘intern’, ‘foundation doctor’, ‘resident’, ‘trainee doctor’, ‘doctor in training’ and ‘house officer’. In the present study, the term house officer (HO) has been used to refer to junior doctor working in the Malaysian healthcare system. A multicentre cross-sectional study was conducted in 12 government hospitals accredited with housemanship training (GHAHT) within the central zone of Malaysia. The main reason for selecting GHAHT was to enable the sampling of HO. Meanwhile, the central zone of Malaysia was selected because it is the most populated zone and houses the largest number of GHAHT, as well as all types of GHAHT, including state, major specialist, university and military hospitals (22).

Following ethical approval, permission to conduct the study was granted for 12 of the 16 GHAHT located within the central zone of Malaysia. HO who were working for at least 6 months in the general medicine, general surgery, orthopaedic, obstetrics and gynaecology, paediatrics, emergency medicine and anaesthesiology departments of these hospitals were universally sampled. A 6-month clinical experience cut-off was chosen as workplace bullying is described to be a persisting phenomenon in which exposure to negative actions have had occurred for at least 6 months (23–24). Those who declined to participate in the study, did not return their questionnaires and on maternity or medical leaves were excluded from the study. A priori sample size calculation based on an estimated 4,991 population of house officers in Malaysia (22) and bullying prevalence of 14% among junior doctors identified from previous studies (25) was determined to be 215, using the OpenEpi calculator (version 3).

### Data Collection

Data was collected in several ways. In the first study site, an email survey was conducted due to its ease of administration, extensive coverage and flexibility in choosing when to complete the questionnaire. The study questionnaire was recast in Google Form format, with a participant information sheet and electronic consent form attached to the first part. It was designed to be anonymous and this was emphasised to study participants. An email including a confidential link to the Google Form was sent to all HOs in the mailing list retrieved from the administration unit of the clinical departments. A week later, reminder to complete the study questionnaire was carried out using email reminders, verbal reminders from group supervisors and individual text messaging. Though no emails were reported to bounce back, the response rate from the email survey was poor. Thus, for the remaining study sites, data was collected via a self-administered paper questionnaire. Again, the study questionnaire was designed to be anonymous and this was emphasised to the participants. In addition, completed questionnaires were asked to be kept in a brown opaque envelope and separate from the participant information sheet and consent form. The data collection was conducted in two phases. For each hospital, the list of HO was first retrieved from the Human Resource Department, whereas information on the date and time of meetings involving HO was obtained from the Occupational Safety and Health officer. In the first phase, the principal investigator invited all HOs present in the meeting to participate in the study. Those who agreed were asked to complete the study questionnaire and return it by the end of the meeting. Those who were not present in the meeting were identified from the attendance list and later included in a new list. In the second phase, the new list was then given to the HO team leaders of each department, who were asked to help distribute the study questionnaires to their colleagues. The principal investigator then collected the questionnaires from the team leaders after two weeks. The entire process of data collection continued for almost 6 months, from 27 November 2017 to 17 May 2018.

### Instruments

Single item questions were used to measure sociodemographic characteristics such as age, gender, ethnicity, educational background and English proficiency as well as employment characteristics such as working duration, medical specialty and type of hospital. In relation to this, educational background was assessed by a single item question asking study participants to state which medical school they graduated from, whereas English proficiency was evaluated using a self-reported item, “How well do you think you speak English?”, with response options ranging from ‘excellent’, ‘good’, ‘fair’ and ‘poor’. The Negative Acts Questionnaire-Revised (NAQ-R) and a stem question based on a definition of workplace bullying were used to measure workplace bullying. The NAQ-R is a 22-item scale that measures study participants’ exposure to work-related bullying, person-related bullying and physical intimidation within the past 6 months, with study participants rating their exposure as ‘Never’, ‘Now and then’, ‘Monthly’, ‘Weekly’ and ‘Daily’ (2). Post-hoc validation demonstrated a one-factor solution and excellent reliability (intraclass correlation coefficient [ICC] of 0.94 and Cronbach’s alpha of 0.97). The NAQ-R was followed up by the stem question asking study participants whether they perceive being bullied at work, based on the following definition of workplace bullying: ‘Workplace bullying refers to situations where an employee is persistently exposed to negative and aggressive behaviour at work primarily of a psychological nature with the effect of humiliating, intimidating, frightening or punishing the target’ (2). It has the same response anchors as the NAQ-R. Following that, the study participants were asked to answer a single item question to select the commonest perpetrators of negative actions at the workplace.

### Data Analysis

Statistical analyses were performed using the Software for Statistics and Data Science (STATA) version 14.0. Initial data analysis included assessment of missingness and influential data, as well as model checking for logistic regression. Model diagnostics indicated that all assumptions were met as: i) the dependent variable was binary, which was the appropriate structure for logistic regression; ii) multicollinearity analysis indicated that all variables had acceptable variance inflation factor (VIF < 10) and tolerance (tolerance > 0.1) and iii) residual-versus-fitted plot showed that linearity in the transformed expectations was observed. In relation to missingness, results suggested that missingness for study instrument items was less than 5% and also missing completely at random, and thus ignorable. Sensitivity analysis was also conducted to determine whether there was any difference in the outcome according to study site or mode of data collection. No significant differences in prevalence of bullying according to data collection method or hospital were observed and thus, data was pooled together for subsequent analyses.

Next, data were categorised. Educational background was categorised by region, i.e. local, Western Europe, Eastern Europe, Australasia, Middle East, East Asia, South Asia and Southeast Asia. Medical specialty group was categorised according to the classification of medical specialties, which included medical (general medicine and paediatrics), surgical (general surgery and orthopaedic surgery) and mixed (obstetrics and gynaecology, emergency medicine, and anaesthesiology) specialties (26). Following that, to describe the characteristics of study participants, prevalence and types of workplace bullying experienced by study participants, and commonest perpetrator of negative actions, descriptive statistics was conducted using mean and standard deviations for continuous data and frequency and percentage for categorical data. The prevalence of workplace bullying was calculated using the following methods that were derived from literature review: i) Behavioural Experience Method and NAQ-R cut off scores (27); ii) Behavioural Experience Method and Leymann’s Criterion (23, 28); iii) Behavioural Experience Method and Mikkelsen and Einarsen’s Criterion (29); iv) Self-labelling with Definition Method (24) and v) Combination Method based on Both Behavioural Experience and Self-labelling Methods (4). In this study, cases of bullying was operationalised as scoring 45 or more on the NAQ-R, in line with the recommended cut-off score proposed by Notalaers and Einarsen (27), as well as perceiving to be bullied weekly or daily according to the definition of bullying given. The frequency of negative actions experienced was categorised into: i) never, now and then or monthly and ii) weekly or daily, to denote infrequent and frequent exposures respectively. To investigate the possible association between all factors and workplace bullying, simple logistic regression analysis was performed and the crude odds ratio (COR) and 95% confidence interval (CI) was estimated. Variables that were significant at a *P*-value of less than 0.25 were entered into a multiple logistic regression analysis to predict the final independent factors, and the adjusted odds ratio (AOR) and 95% CI was estimated. Additionally, model fitness was assessed using the Hosmer-Lemeshow goodness-of-fit test, with a *P*-value of less than 0.05 taken as an indication of poor fit.

## Results

The overall response rate of this study was 62% (*n* = 1,074). The characteristics of study participants are outlined in Table 1. The study participants had a mean age of 27.0 ± 1.5 and were mainly composed of female participants (65%). In terms of ethnicity, Malay participants comprised the majority (67%) and most of the study participants graduated from medical schools in Malaysia (52%), followed by medical schools in countries within the Middle East (17%) and Eastern Europe (10%). Majority of study participants rated their English-speaking proficiency as good (54%). The study participants had a mean duration working of 15.5 ± 7.0 months and were evenly distributed across all medical specialties (ranging from 31% to 35%). Study participants from major specialist hospital contributed to the majority of the study sample (63%), followed by state hospital (26%) and university hospital (11%).

The prevalence of workplace bullying among study participants was 13% (Table 2). The most frequent types of negative actions reported by the study participants included work-related bullying such as ‘being ordered to do work below your level of competence’ (21% weekly or daily), person-related bullying such as ‘being humiliated or ridiculed in connection with your work’ (17% weekly or daily), and physically intimidating bullying such as ‘being shouted at or being target of spontaneous anger’ (16% weekly or daily) (Table 3). The commonest perpetrators of negative actions at work were reported to be medical officers (59%), followed by nurses and support staff (31%) (Table 4).

In the univariate analysis, significant associations were observed between ethnicity, education region, specialty group and workplace bullying (Table 5). After controlling for potential confounders, education region, proficiency in English and specialty group emerged as significant predictors of workplace bullying among study participants. Study participants who graduated from Eastern European medical schools (AOR 2.27; 95% CI: 1.27, 4.07) and worked in surgical-based rotation (AOR 1.83; 95% CI: 1.13, 2.97) had higher odds of workplace bullying compared to those who graduated from local medical schools and worked in medical-based rotation. On the other hand, study participants with good English proficiency (AOR 0.14; 95% CI: 0.02, 0.94) had lower odds of workplace bullying compared to those with poor English proficiency. No differences in bullying were observed in relation to age, gender, ethnicity, working duration and type of hospital.

## Discussion

The present study examined the prevalence of workplace bullying among Malaysian junior doctors and explored the sociodemographic and employment factors associated with it. Based on the analysis of a universal sample of junior doctors sampled across 12 government hospitals accredited for housemanship training within the central zone of Malaysia (*n* = 1,074), it was observed that the 6-month prevalence of workplace bullying among junior doctors was 13%. This is comparable to the study published by Ling et al. (25), who reported that 14% of junior doctors were exposed to workplace bullying on a weekly or daily basis for the past 12 months. The systematic review of prior studies on the prevalence of workplace bullying among junior doctors has suggested that a wide range of prevalence (30%–95%) of bullying have been reported, depending on the study operationalisation of bullying (6). This may be partly explained by the heterogeneity in the terms and methodologies used to examine workplace bullying as well as definitional issues in relation to the persistence of negative interactions experienced (6). Indeed, methods used by researchers to measure workplace bullying vary, which include the Behavioural Experience Method using a bullying inventory (e.g. NAQ-R) and/or the Self-labelling Method using a bullying definition. Furthermore, even when the same instrument is used, researchers tend to vary in selecting the methods by using either the Leymann criterion (23, 28), Mikkelsen and Einarsen’s criterion (29) or the cut-off score. As shown in the present study, different methods of measuring workplace bullying among the same study population yielded different prevalence (16%–43%) and more stringent operational definition resulted in lower prevalence of workplace bullying. Thus, the combination method was considered to be the most appropriate method to measure workplace bullying, as it could capture both the persistency of negative actions experienced by the study participants as well as their subjective interpretation of being victimised (4, 24, 29).

Further, despite the combination method being a more conservative measurement method, a prevalence of 13% indicated that a significant proportion of Malaysian junior doctors experience bullying at work. This is evident from the results of meta-analysis reviewing a wide selection of studies (*n* = 44,878, *k* = 15 samples), which showed that the combination method led to a weighted prevalence of 3.7% bullying (4), suggesting that junior doctors were relatively more frequent targets of bullying compared to the general working population. In relation to this, contextual factors which are unique to junior doctors such as strict medical hierarchy, especially demanding, fast-paced and unpredictable work, and widespread custom of ‘teaching by humiliation’ during medical training (8, 21) may have partly contributed to this observation. In terms of geographical variation in the prevalence of workplace bullying among junior doctors, no stark contrast was observed from the findings of Samsudin et al. (6), though it should be noted that methodological heterogeneity often acts as an impediment for objective comparisons. Nonetheless, given homogeneity in such aspects, it would have been interesting to relate differences in bullying to contextual dissimilarities inherent in different junior doctor populations. This is because junior doctors in different parts of the world are subject to variable resident duty hours, environment for training and amount of clinical supervision (30), and that there is evidence of cross-cultural differences between countries within and across a region (31).

In terms of types of negative actions experienced, the findings of this study indicated that junior doctors experienced all types of bullying, including work-related, person-related and physically intimidating bullying. In comparison, the findings of Ling et al. (25), who also used the NAQ-R, indicated that junior doctors reported higher exposure to work-related bullying compared to person-related and physically intimidating bullying. In addition, contrary to this study, which reported that medical officers (58%) followed by nurses and support staff (31%) were the commonest perpetrator of negative actions at the workplace, the commonest perpetrators reported by Ling et al. (25) were consultants (54%), followed by administration (28%) and fellow trainees (13%).

The findings of this study suggested that the probability of workplace bullying among junior doctors was higher in certain medical specialties, as study participants working in surgical-based rotation had 83% higher odds of bullying compared to those working in medical-based rotation. Correspondingly, Dikmetas et al. (18) and Al-Shafaee et al. (10) also reported significant differences in negative interactions experienced by junior doctors according to medical specialties. In relation to this, Dikmetas et al. (18) indicated that junior doctors perceived negative interactions more frequently in surgical medicine compared to basic medicine and internal medicine (*P* = 0.001), whereas Al-Shafaee et al. (10) reported that mistreatment occurred more commonly during medical rotation compared to surgical and paediatric rotation (*P* = 0.005). This suggests that there are differences in terms of job demands and resources and subsequent job strain between the medical specialties, which can be explained by the Job Demands-Resources Model (32). Indeed, certain medical specialties have been suggested to demand more time and provide less emotionally and socially supportive working environments (19), and it is evident from existing literature that job demands relate positively to targets’ reports of bullying, whereas job resources relate negatively to bullying (32).

Besides that, other significant predictors of workplace bullying among junior doctors include their educational background and proficiency in English language. It was observed that study participants who graduated from Eastern European medical schools had twice the odds of bullying at work compared to those who graduated from local medical schools. Although there is no evidence to support this observation presently, it may be postulated to be due to the differences in the level of confidence and preparedness for hospital work between junior doctors graduating from traditional and nontraditional medical programmes (33). Until now, Eastern European medical schools have been teaching their disciplines traditionally and have not yet fully adopted the Integrated Pre-clinical Medical Education Programme (34). According to the study published by Eley (33), junior doctors who spent undergraduate years training at non-traditional medical schools felt more confident and better equipped for internship. Thus, it may be that those who graduated from medical schools within the region were less assured in managing daily tasks compared to their peers, which may have led them to be vulnerable targets for frequent bullying at the workplace.

On the other hand, English language proficiency was shown to be a protective factor for junior doctors, as study participants who rated their English proficiency as good had 86% lower odds of workplace bullying compared to those who rated their English proficiency as poor. Though there are no studies to support this finding, the underlying reason could be the fact that those with poor English proficiency may be less adept communicators. This may have caused them to be more frequently misconstrued by others, generating negative reactions among their co-workers. Indeed, in the qualitative study conducted by Marzuki et al. (35) to assess the congruence between language proficiency and communicative abilities, study participants reported that those with poor communicative abilities were those who had poor command over the language.

The present study also demonstrated no significant differences between bullied and non-bullied participants with regards to age, gender, ethnicity, working duration and type of hospital. However, several studies conducted elsewhere have indicated that age, gender and ethnicity are significantly associated with workplace bullying among junior doctors. In relation to age, Crutcher et al. (36), Chadaga et al. (37), Bairy et al. (38), Scott et al. (39) and Hills et al. (40) observed significant differences in negative interactions experienced according to age, with those younger than 30 years of age being more likely to experience workplace bullying. This may be because there is a traditional power structure and hierarchy within the medical setting in which junior doctors, who are typically younger, are at the lowest end of the pecking order (36), making them more prone to bullying. On the other hand, it has also been postulated that with increasing age and maturity, perceptions and interpretations change, which may be the reason why older doctors are less likely to perceive bullying (36). As only House Officers were sampled in this study, the difference in the baseline age is small, which may explain the non-significant finding.

In terms of gender, studies published by Chadaga et al. (37), Aykut et al. (17), Fnais et al. (41), Ling et al. (25) and Hills et al. (40) consistently reported that significantly more female junior doctors experience bullying at work compared to their male counterparts. This was believed by some to be due to men and women perceiving workplace bullying differently, with men being more likely to perceive bullying as a particular management style, whereas women being more likely to perceive it as threatening (42). Others argue that women are permitted narrower bands of acceptable behaviour, and consequently, deviations from traditional roles may submit them to negative evaluations and increase their risk of experiencing workplace bullying (43–44). Finally, in relation to ethnicity, Chadaga et al. (37) reported significant differences in workplace bullying among white and non-white participants. This difference could perhaps be attributed to inequalities in both personal and social vulnerabilities among employees of different ethnicities that are intrinsic in certain cultures (45).

Though working duration was not a significant predictor of bullying among junior doctors, it may influence their exposure to bullying because of the nature of bullying itself, which involves a perceived power imbalance (2). This is because those who have worked for shorter durations are more likely to have less knowledge and skills compared to their seniors, making them more prone to workplace victimisation. Furthermore, it has been suggested that junior doctors are less skilled and experienced in minimising and deescalating conflicts at the workplace (40), making them more susceptible to frequent bullying, which is a notion that is more likely for those with less working duration.

The present study has several limitations. The multicentre cross-sectional study design, even though considered a cost-effective and practical approach, is not able to establish evidence for causality. In addition, workplace bullying is described as a process that often progresses and escalates over time (46); as the study was conducted among junior doctors that were currently employed, those who had been severely affected by bullying to the extent that they had resigned or developed illness requiring long-term leave may have been excluded from the study. As such, the prevalence of workplace bullying may have been underestimated. Besides that, though the generalisability of this study was improved by applying universal sampling as well as achieving a response rate of 62%, which is higher than the average response rate for surveys used in organisational research (47), a nonresponse of approximately 40% may have influenced the representativeness of the findings. Another limitation stems from the use of self-administered questionnaire, which could have led to several biases, including recall bias and social desirability bias. In this regard, social desirability bias may have been possible as bullying often goes unreported because targets of bullying feel ashamed (48) and may have chosen to report otherwise to avoid perceiving themselves as victims. However, it may have been reduced as participant anonymity and study confidentiality were emphasised during the recruitment process. On the other hand, recall bias was unavoidable as the questionnaires were anonymous and there was no method of contacting study participants to verify any irregular responses once they have submitted their questionnaires.

Despite the above limitations, several steps were taken to increase the robustness of the present study, including sampling from multiple study sites and applying universal sampling procedures to increase external validity of study findings, conducting a priori sample size calculation to ensure adequate study power, and using validated instrument that was further verified via post-hoc exploratory factor analysis and reliability testing as well as adjusting for potential confounders in the final model to improve the internal validity of study findings.

## Conclusion

The findings of the present study indicate that workplace bullying is a significant issue for Malaysian junior doctors and that at least 1 in 10 Malaysian junior doctors perceives being bullied at work. Considering that bullying is an underreported problem, this is worrying. The findings of the present study also suggest that certain sociodemographic and employment characteristics may be predictive of junior doctors’ exposure to workplace bullying. This implies a need to perform risk-stratification to identify junior doctors who are most at risk of experiencing bullying at work so that early, targeted interventions can be initiated. Indeed, the considerable costs of workplace bullying should justify that tackling it be made a priority. In fact, according to Sheehan (49), the costs of workplace bullying prevention strategies are marginal compared to the costs of workplace bullying on organisations. To facilitate this, future studies examining workplace bullying among junior doctors should include prospective studies that explore antecedents of bullying, in order to explore remedial actions. In this regard, studies published by Samsudin et al. (50) and Samsudin et al. (51) have shown that individual traits such as negative affect and neuroticism, as well as organisational characteristics including organisational climate, culture, leadership, justice, and support, are significant factors of workplace bullying among junior doctors. However, causality has not been established for these factors. Further, variables associated with workplace bullying such as psychological capital (52), social, coping and problem-solving skills (53–59), conflict management styles (57), core self-evaluations (60), organisational change (61–63), and societal norms and culture (64–66) have also not been examined among junior doctors. Given the seriousness of the consequences in relation to junior doctors’ ability to learn and provide safe patient care, greater awareness of workplace bullying in medicine should be given utmost importance and healthcare policies to tackle this occupational hazard should be developed and implemented promptly.

## Figures and Tables

**Table 1:** Characteristics of study participants (*N* = 1,074)

Variable	Mean ± SD	*n* (%)
Age (years)	27.0 ± 1.5	
Gender		
Male		371 (34.6%)
Female		701 (65.4%)
Ethnicity		
Malay		710 (66.5%)
Chinese		159 (14.9%)
Indian		180 (16.9%)
Others		18 (1.7%)
Academic graduation by region		
Local		546 (52.4%)
Western Europe		56 (5.4%)
Eastern Europe		104 (10.0%)
Australasia		14 (1.3%)
Middle East		181 (17.4%)
East Asia		2 (0.2%)
South Asia		56 (5.4%)
Southeast Asia		83 (8.0%)
English proficiency		
Poor		5 (0.5%)
Fair		284 (26.9%)
Good		567 (53.6%)
Excellent		201 (19.0%)
Duration working (months)	15.5 ± 7.0	
Medical specialty group		
Medical		356 (34.6%)
Surgical		318 (30.9%)
Mixed		354 (34.4%)
Type of hospital		
State hospital		281 (26.2%)
Major specialist hospital		675 (62.9%)
University hospital		118 (11.0%)

**Table 2:** Prevalence of workplace bullying among participants (*N* = 1,074)

Method of measuring workplace bullying	Bullied	

	No	Yes
Persistent exposure to negative actions, i.e. scoring > 45 on NAQ-R^A^ (*n* = 1,041)	644 (61.9%)	397 (38.1%)
Persistent exposure to negative actions, i.e. exposure to at least one negative action on a weekly or daily basis for the past 6 months^B^ (*n* = 1,041)	588 (56.5%)	453 (43.5%)
Persistent exposure to negative actions, i.e. exposure to at least two negative actions on a weekly or daily basis for the past 6 months^C^ (*n* = 1,041)	688 (66.1%)	353 (33.9%)
Perceive to be bullied according to bullying definition given D (*n* = 1,042)	878 (84.3%)	164 (15.7%)
**Prevalence of workplace bullying**^E^ (*n* = 1,025)	889 (86.7%)	136 **(13.3%)**

Notes:

A based on behavioural experience method and NAQ-R cut-off

B based on behavioural experience method and scores; Leymann’s criterion;

C based on behavioural experience method and Mikkelsen and Einarsen’s criterion;

D based on self-labelling with definition method;

E based on combination method (methods A and D)

**Table 3:** Types of workplace bullying experienced by participants (*N* = 1,074)

Types of negative actions	Never, now and then or monthly *n* (%)	Weekly or daily *n* (%)
1. Someone withholding information which affects your performance	959 (90.8%)	97 (9.2%)
2. Being humiliated or ridiculed in connection with your work	881 (83.4%)	175 (16.6%)
3. Being ordered to do work below your level of competence	833 (79.2%)	219 (20.8%)
4. Having key areas of responsibility removed or replaced with more trivial or unpleasant tasks	884 (84.8%)	171 (16.2%)
5. Spreading gossip about you	914 (86.6%)	141 (13.4%)
6. Being ignored or excluded	945 (89.6%)	110 (10.4%)
7. Having insulting or offensive remarks made about your person, attitudes, or private life	942 (89.3%)	113 (10.7%)
8. Being shouted at or being target of spontaneous anger	885 (83.9%)	170 (16.1%)
9. Intimidating behaviours such as finger-pointing, invasion of personal space, shoving, blocking your way	922 (87.5%)	132 (12.5%)
10. Hints or signals from others that you should quit your job	978 (92.5%)	79 (7.5%)
11. Repeated reminders of your errors or mistakes	926 (87.6%)	131 (12.4%)
12. Being ignored or facing a hostile reaction when you approach	946 (89.5%)	111 (10.5%)
13. Persistent criticism of your work and effort	932 (88.3%)	124 (11.7%)
14. Having your opinions ignored	939 (88.8%)	118 (11.2%)
15. Practical jokes carried out by people you do not get along with	967 (91.5%)	90 (8.5%)
16. Being given tasks with unreasonable deadlines	944 (89.3%)	113 (10.7%)
17. Having allegations made against you	986 (93.4%)	70 (6.6%)
18. Excessive monitoring of your work	947 (89.9%)	107 (10.1%)
19. Pressure to not claim something to which by right you are entitled to	896 (85.0%)	158 (15.0%)
20. Being the subject of excessive teasing and sarcasm	953 (90.3%)	103 (9.7%)
21. Being exposed to unmanageable workload	904 (85.5%)	153 (14.5%)
22. Threats of violence or physical abuse or actual abuse	1,001 (94.7%)	56 (5.3%)

**Table 4:** Commonest perpetrator of negative actions reported by participants (*N* = 1,074)

Source	Perpetrator of negative actions	

	No	Yes
Consultants and specialists	782 (72.8%)	292 (27.2%)
Medical officers	439 (40.9%)	635 (59.1%)
House officers	890 (82.9%)	184 (17.1%)
Nurses and support staff	741 (69.0%)	333 (31.0%)
Administrative and non-clinical staff	1,030 (95.9%)	44 (4.1%)

**Table 5:** Association of sociodemographic and employment factors with workplace bullying (*N* = 1,025)

Variables	COR (95% CI)^A^	*P-*value	AOR (95% CI)^B^	*P-*value
Age	0.91 (0.79, 1.04)	0.156	0.91 (0.78, 1.07)	0.239
Gender			n/s	
Male	1.00 (ref)			
Female	0.83 (0.57, 1.21)	0.344		
Ethnicity				
Malay	1.00 (ref)		1.00 (ref)	
Chinese	1.08 (0.63, 1.85)	0.772	1.00 (0.54, 1.85)	0.996
Indian	**1.72 (1.10, 2.69)**	**0.017**	1.69 (0.97, 2.93)	0.063
Others	1.50 (0.43, 5.31)	0.526	1.76 (0.46, 6.67)	0.408
Education region				
Local	1.00 (ref)		1.00 (ref)	
Western Europe	1.46 (0.68, 3.13)	0.333	2.10 (0.94, 4.69)	0.069
Eastern Europe	2.04 (1.18, 3.50)	0.010	2.27 (1.27, 4.07)	0.006
Australasia	1.19 (0.26, 5.43)	0.825	1.57 (0.32, 7.69)	0.578
Middle East	0.87 (0.51, 1.50)	0.625	1.15 (0.61, 2.14)	0.670
East Asia	1.00*		1.00*	
South Asia	0.89 (0.37, 2.16)	0.798	0.80 (0.30, 2.17)	0.664
Southeast Asia	0.67 (0.30, 1.53)	0.344	0.83 (0.35, 1.97)	0.680
English proficiency				
Poor	1.00 (ref)		1.00 (ref)	
Fair	0.24 (0.04, 1.49)	0.125	0.20 (0.03, 1.37)	0.101
Good	0.19 (0.03, 1.17)	0.074	**0.14 (0.02, 0.94)**	**0.043**
Excellent	0.31 (0.05, 1.95)	0.213	0.21 (0.03, 1.44)	0.112
Duration working	0.98 (0.95, 1.01)	0.122	0.99 (0.96, 1.02)	0.606
Specialty group				
Medical	1.00 (ref)		1.00 (ref)	
Surgical	1.78 (1.13, 2.81)	0.012	1.83 (1.13, 2.97)	0.014
Mixed	1.00 (0.62, 1.64)	0.989	1.10 (0.64, 1.88)	0.737
Type of hospital			n/s	
SH	1.00 (ref)			
MSH	0.82 (0.55, 1.23)	0.346		
UH	0.69 (0.35, 1.37)	0.291		

Notes: SH = state hospitals; MSH = major specialist hospitals; UH = university hospitals; n/s = non-significant;

A COR estimates from simple logistic regression;

B AOR estimates from multiple logistic regression;

* Insufficient sample size, cell empty; Assumptions of logistic regression have been met and the Hosmer-Lemeshow goodness-of-fit test indicated good fit (*P*= 0.558)
